# Caspase-2 and p75 neurotrophin receptor (p75NTR) are involved in the regulation of SREBP and lipid genes in hepatocyte cells

**DOI:** 10.1038/s41419-019-1758-z

**Published:** 2019-07-11

**Authors:** Dan Duc Pham, Céline Bruelle, Hai Thi Do, Ceren Pajanoja, Congyu Jin, Vignesh Srinivasan, Vesa M. Olkkonen, Ove Eriksson, Matti Jauhiainen, Maciej Lalowski, Dan Lindholm

**Affiliations:** 1 Medicum, Department of Biochemistry and Developmental Biology, Medical Faculty, University of Helsinki, POB 63, FI-00014 Helsinki, Finland; 2 Minerva Foundation Institute for Medical Research, Biomedicum 2, Tukholmankatu 8, FI-00290 Helsinki, Finland; 3 HiLiFE, Meilahti Clinical Proteomics Core Facility, Helsinki, Finland

**Keywords:** Lipid signalling, Metabolic disorders

## Abstract

Lipid-induced toxicity is part of several human diseases, but the mechanisms involved are not fully understood. Fatty liver is characterized by the expression of different growth and tissue factors. The neurotrophin, nerve growth factor (NGF) and its pro-form, pro-NGF, are present in fatty liver together with p75 neurotrophin receptor (p75NTR). Stimulation of human Huh7 hepatocyte cells with NGF and pro-NGF induced Sterol-regulator-element-binding protein-2 (SREBP2) activation and increased Low-Density Lipoprotein Receptor (LDLR) expression. We observed that phosphorylation of caspase-2 by p38 MAPK was essential for this regulation involving a caspase-3-mediated cleavage of SREBP2. RNA sequencing showed that several genes involved in lipid metabolism were altered in p75NTR-deficient mouse liver. The same lipogenic genes were downregulated in p75NTR gene-engineered human Huh7 cells and reciprocally upregulated by stimulation of p75NTRs. In the knock-out mice the serum cholesterol and triglyceride levels were reduced, suggesting a physiological role of p75NTRs in whole-body lipid metabolism. Taken together, this study shows that p75NTR signaling influences a network of genes involved in lipid metabolism in liver and hepatocyte cells. Modulation of p75NTR signaling may be a target to consider in various metabolic disorders accompanied by increased lipid accumulation.

## Introduction

Many metabolic diseases are characterized by the increased levels of fatty acids (FA) and other lipids that in the long-run may lead to enhanced lipid-induced toxicity (lipoapoptosis)^1^. In fatty liver there is an accumulation of lipids that in humans can cause non-alcoholic fatty liver disease (NAFLD)^2^. The underlying mechanisms are not fully understood but include changes in cell signaling, increased oxidative and ER stress, mitochondrial dysfunctions, caspase activation, and enhanced tissue inflammation with the production of various cytokines and growth factors ultimately leading to increases in certain lipids such as ceramides^3–5^.

Neurotrophins, including nerve growth factor (NGF), are important regulators of neuronal differentiation and survival, but their functions in cell metabolism are only emerging^6–8^. NGF and its pro-form pro-NGF stimulate p75 neurotrophin receptor (p75NTR) that is expressed by particular neurons^9,10^ as well as by some non-neuronal cells including hepatocytes^8,11,12^. We have reported that NGF and pro-NGF are increased in fatty liver of genetically obese *ob/ob* mice, suggesting a role of these neurotrophins in lipid metabolism^8^. In man, higher levels of NGF in plasma have been correlated to obesity and the presence of metabolic syndrome in women^12^, but the underlying mechanisms are not fully understood.

We have previously shown that treatment with NGF/pro-NGF can activate the transcription factor, sterol-regulator-element-binding protein-2 (SREBP2), involved in regulation of lipid and cholesterol-associated genes in human Huh7 hepatocyte cells^8^. Here we have further investigated the mechanisms of SREBP activation by p75NTRs, demonstrating that p38 MAPK and cell caspases are essentially involved in SREBP2 regulation, acting downstream of p75NTRs. In p75NTR gene deleted (p75NTR KO) mice the expression of several genes involved in lipid metabolism was altered as shown by RNA sequencing (RNA-seq), suggesting a role of p75NTR in lipid signaling. In support of this, human Huh7 cells with loss of p75NTR after CRISPR/Cas9-mediated gene editing demonstrated corresponding changes in lipid-associated gene expression. Together, our results show that p75NTR plays a role in the regulation of lipid genes and their expression and thus contributes to lipid-associated metabolic disorders.

## Results

### p75 neurotrophin receptor is expressed in fatty liver together with NGF and pro-NGF

**Fig. 1:**
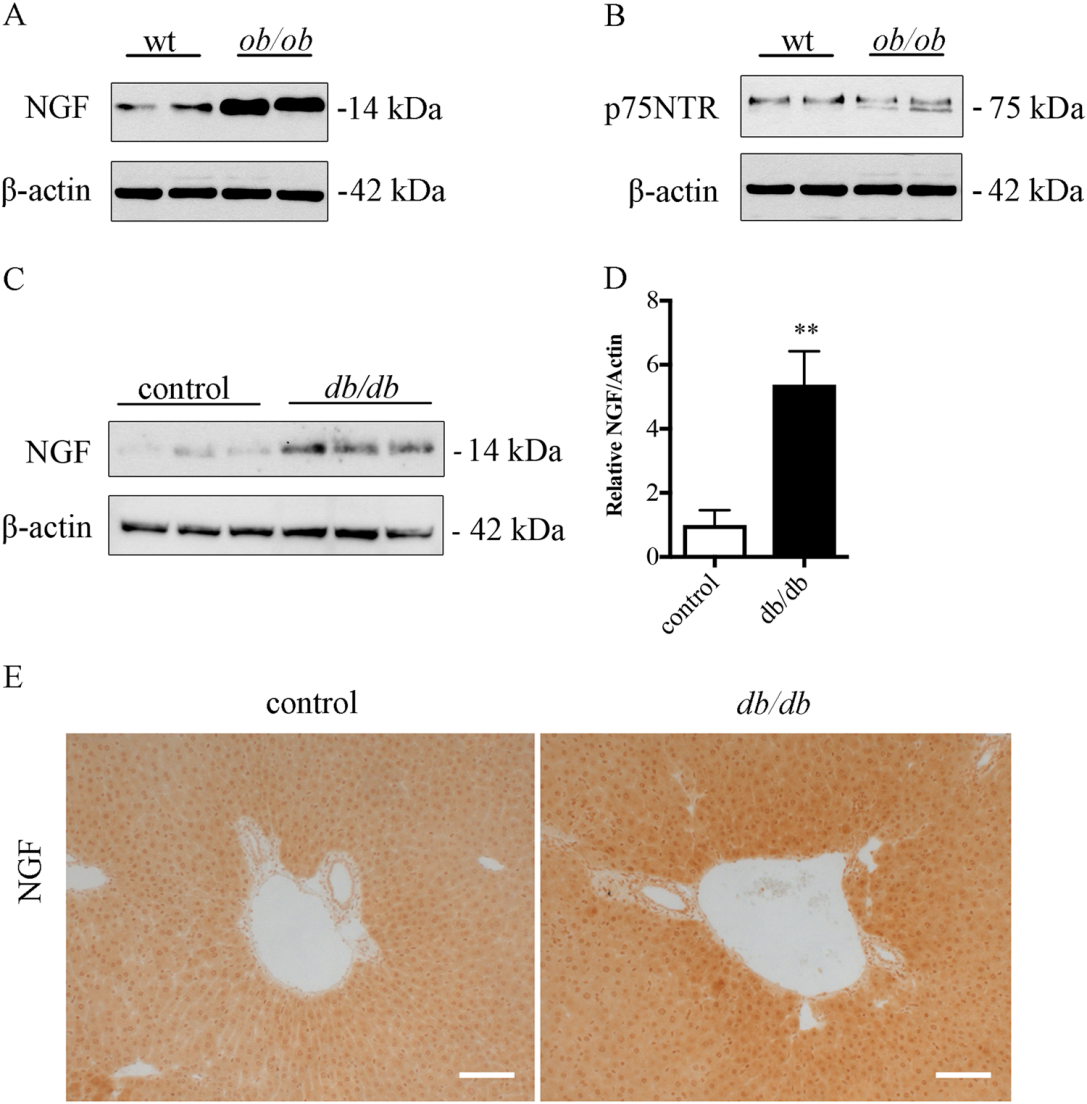
NGF is expressed in fatty liver of obese mice together with p75NTR. **a** NGF has increased in leptin-deficient *ob/ob* mouse livers compared with control, wild-type (wt) mice. **b** p75NTR was expressed in liver with no significant change between *ob/ob* and control mice. **c**, **d** The level of NGF has increased in leptin-receptor-deficient *db/db* mouse liver in comparison to controls, **c** immunoblot analysis and **d** quantification. **p < 0.01 for *db/db* vs. controls. **e** Immunohistochemistry of NGF in control and *db/db* mouse liver. NGF immunoreactivity was present in hepatocytes and in cells adjacent to the liver sinusoids. Scale bar, 100 μm. Liver tissues from control and genetically obese mice were processed for immunoblotting and immunohistochemistry as described in Materials and methods. For immunoblotting, β-actin was used as a loading control

The p75NTR is part of a large cytokine receptor family, which includes the receptors for the pro-inflammatory cytokine, tumor necrosis factor-α. Immunoblots revealed that NGF and p75NTR were expressed in livers of control and obese leptin-deficient *ob/ob* mice (Fig. 1a, b). Previously, we have reported that also pro-NGF was increased in *ob/ob* mice as compared to controls^8^. Likewise, NGF was elevated in leptin-receptor-deficient *db/db* mice, used as another model for fatty liver disease (Fig. 1c, d). Immunohistochemistry demonstrated the presence of NGF in hepatocytes and in the cells lining the liver sinusoids in fatty liver (Fig. 1e), comparable to cellular localization displayed by NGF in the liver affected by other diseases^13^. The presence of NGF and pro-NGF and p75NTRs in fatty liver raised questions as to their physiological role in this disorder.

### SREBPs are activated by the neurotrophins in Huh7 hepatocyte cells

**Fig. 2:**
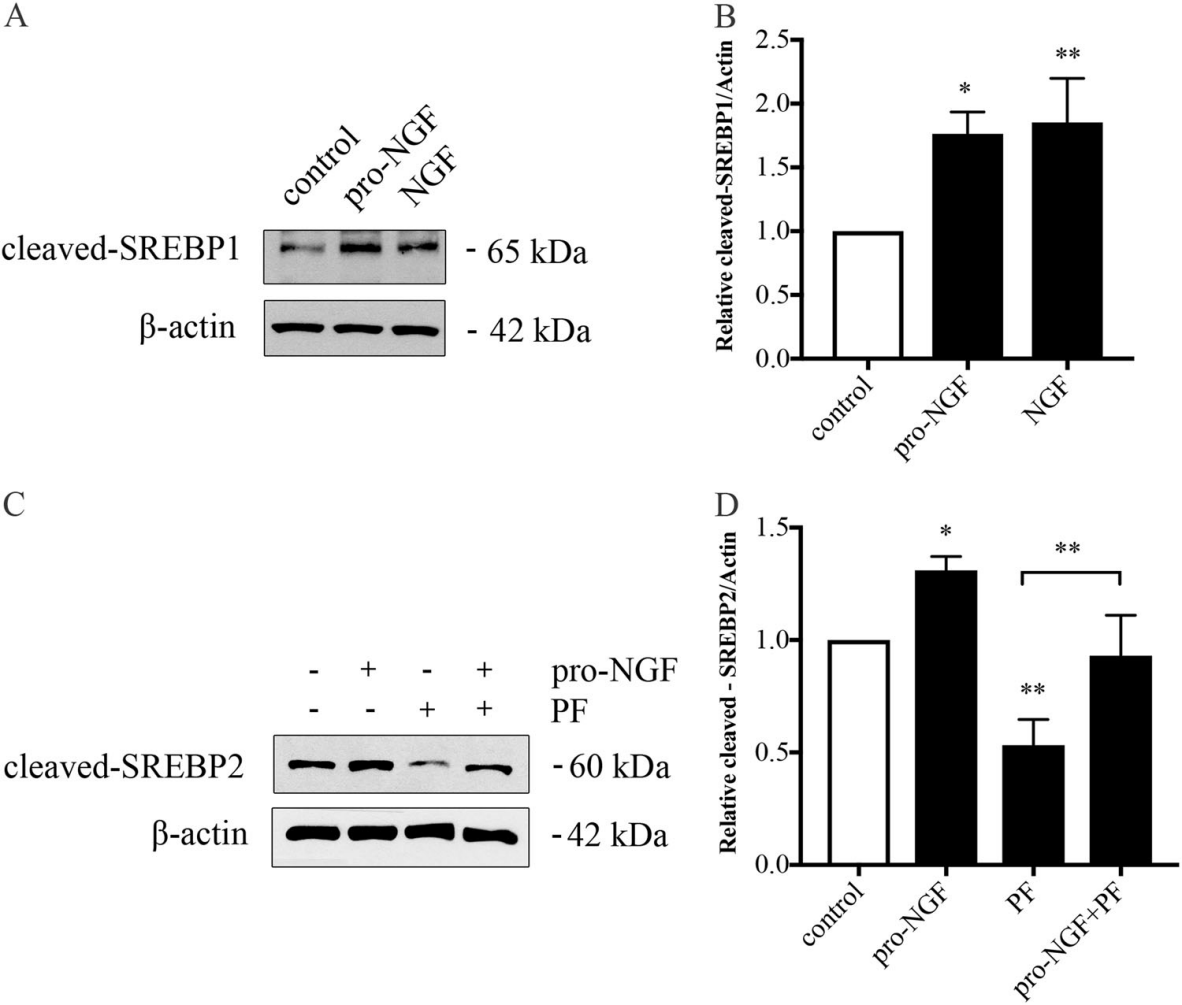
p75NTR stimulation activated SREBP1 and SREBP2 in Huh7 hepatocyte cells. Human Huh7 cells were stimulated with 10 ng/ml pro-NGF or 50 ng/ml NGF for 16 h followed by immunoblotting using antibodies to detect the presence of cleaved/activated SREBP1 and SREBP2. **a** SREBP1, immunoblots and **b** quantification. Values are mean ± SD, *n* = 3. **P < 0.01 and *p < 0.05 for treated vs. control cells. **c**, **d** SREBP2. Cells were treated with 10 ng/ml pro-NGF in the absence or presence of 0.5 μM PF429242 (PF), which is a selective inhibitor of the Site 1 protease (S1P). **c** Immunoblots and **d** quantification. PF decreased the amount of processed SREBP2 in these cells; however, addition of pro-NGF increased the relative level of cleaved SREBP2. Values are mean ± SD, *n* = 3. *p < 0.05 for treated vs. the corresponding control

The SREBP family of transcription factors includes SREBP1a and SREBP1c and SREBP2, which are activated by low cell cholesterol to induce synthesis of genes involved in lipid metabolism^14,15^. Typically, the SREBPs are present in the endoplasmic reticulum in latent forms and can be cleaved by the enzymes, namely site-1 protease (S1P) and site-2 protease (S2P) to yield the active transcription factor^15,16^. In addition to S1P and S2P, the SREBP molecules can be cleaved by caspase-3 (ref. ^17^), but the physiological significance of this cellular event has remained unclear. We previously reported that stimulation of p75NTRs by NGF and pro-NGF induced cleavage of SREBP2 in Huh7 hepatocyte cells in a caspase-3-dependent manner^8^. Treatment of Huh7 cells with NGF/pro-NGF also enhanced the cleavage of SREBP1 (Fig. 2a, b), suggesting that the neurotrophin-mediated regulation is a general phenomenon involving both SREBP1 and SREBP2. To investigate the links between the known S1P-mediated processing of SREBP2 and the cleavage induced by the neurotrophins, we employed the compound PF-429242 (ref. ^18^). The level of processed SREBP2 was reduced in cells treated with PF-429242, which is in line with its recognized action as an inhibitor of S1P (Fig. 2c, d). However, the rise in the SREBP2 cleavage evoked by pro-NGF was largely unaffected in the presence of PF-429242, suggesting that the two SREBP2 processing pathways likely represent different events (Fig. 2c, d).

### Roles of Caspase-2 in SREBP regulation

**Fig. 3:**
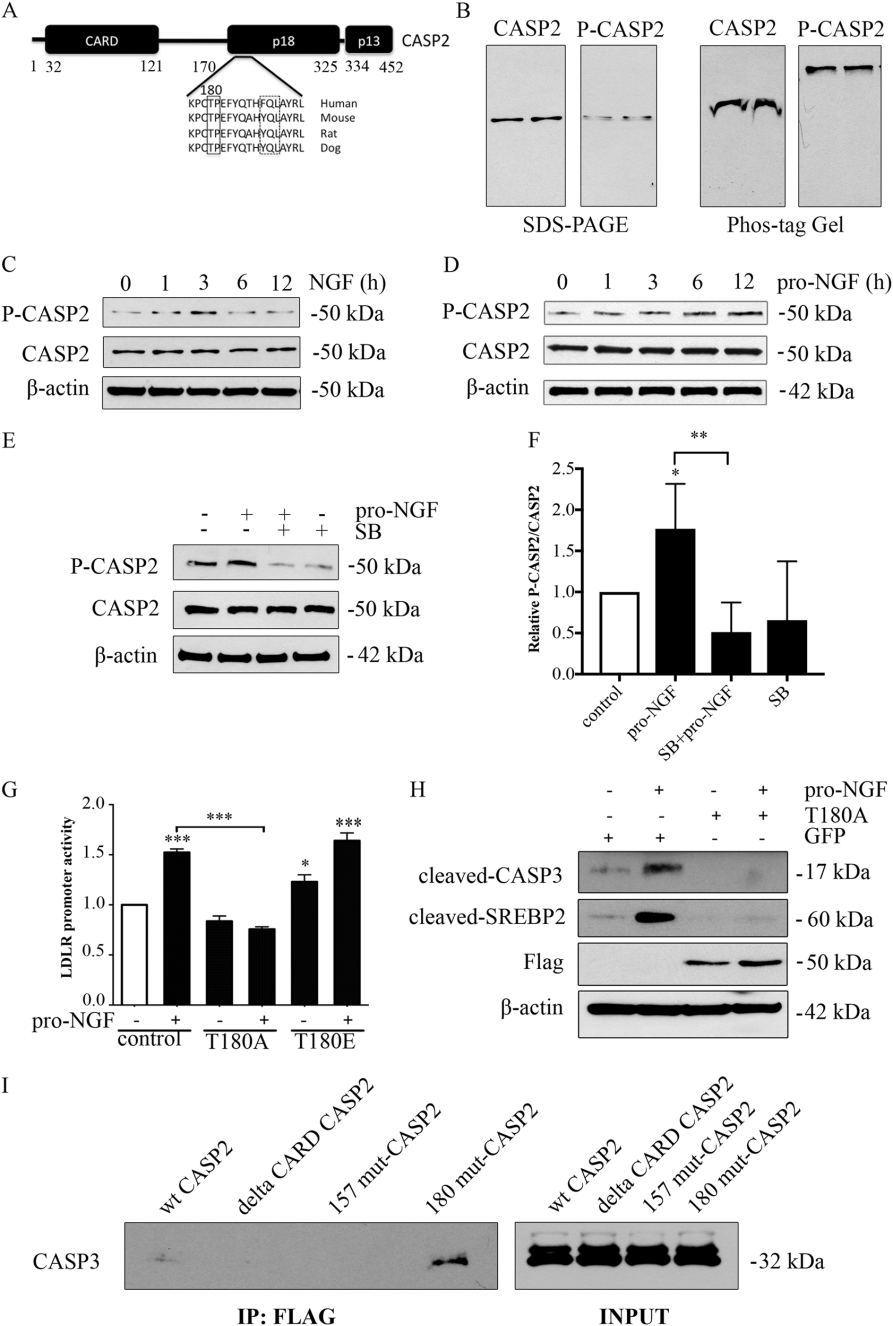
Role of Caspase-2 in p75NTR mediated regulation of SREBP. **a** Structure of human caspase-2 (CASP2) showing the CARD domain and the 18 and 13 kDa caspase subunits. p38 MAPK phosphorylation site at threonine (Thr)180 in CASP2 is conserved among species. The amino acid numbering underlying functional domains in CASP2 is shown. Boxes represent sequence homology among mammalian species. **b** Phosphoprotein retardation was performed as described in Materials and methods. A specific phospho-CASP2 antibody against Thr180 was used in conjunction with an antibody against CASP2, recognizing non-phosphorylated form of the enzyme. Phosphorylated CASP2 migrated slower in the Phos-Tag gel (right) as compared with the protein run on denaturating SDS-PAGE (left). **c**, **d** Huh7 hepatocyte cells were stimulated with 50 ng NGF (**c**) or 10 ng/ml pro-NGF (**d**) for different times followed by immunoblotting using phospho-CASP2 and anti-CASP2 antibodies. β-Actin was used as a control. **e**, **f** Cells were treated with pro-NGF for 6 h in the absence or presence 1 μM of the p38 MAPK inhibitor, SB203580 (SB). **e** Immunoblot and **f** quantification of p-CASP2 levels. SB reduced the increase in p-CASP2 by pro-NGF. Values are means ± SD, *n* = 4. *p < 0.05 for pro-NGF vs. control and **p < 0.01 for SB + pro-NGF vs. pro-NGF. **g** Mutant Flag-Thr180A-CASP2 (T180A) and Flag-Thr180E-CASP2 (T180E) caspase-2 constructs were generated by site-directed mutagenesis and transfected for 24 h into Huh7 cells in conjunction with the LDLR promoter construct to measure gene activity. Control cells expressed EGFP plasmid. Half of the cells was then stimulated with 10 ng/ml pro-NGF for 16 h. Luciferase activity was measured as described and normalized to Renilla readout. Expression of mutant T180A construct inhibited the effect of pro-NGF on LDLR, while the T180E construct increased LDLRs in controls. pro-NGF values are mean ± SD, *n* = 4. ***p < 0.001 for pro-NGF vs. unstimulated controls, and for T180A + pro-NGF vs. pro-NGF. *p < 0.05 for T180E unstimulated vs. controls. **h** Cells transfected with control GFP or mutant T180A-CASP2 construct were further stimulated with 10 ng/ml pro-NGF for 16 h. The amount of cleaved caspase-3 (17 kDa band, CASP3) and of SREBP2 (60 kDa band) was induced by pro-NGF and reduced in the presence of T180A. Expression of T180A construct is shown using anti-Flag antibodies. **i** Cell lysates from wild type and mutant caspase-2-expressing cells were subjected to co-immunoprecipitation (IP) using anti-Flag antibodies as described in Materials and methods. Immunoblotting was done using anti-caspase-3 antibodies. Lane 1, wild-type caspase-2; Lane 2, CARD domain lacking caspase-2 mutant; Lane 3, Ser157A caspase-2 mutant; Lane 4, Thr180A caspase-2 mutant

To clarify the role of caspases in SREBP regulation, we focused on upstream caspase-2, reported to play a role in models of fatty liver disease and in NAFLD^4,19^. We previously demonstrated that caspase-2 and p38 MAPK are both downstream of p75NTR-mediated signaling cascade in human Huh7 cells^8^, raising the question whether caspase-2 may be phosphorylated by this kinase. Bioinformatics analyses revealed that there is a potential phosphorylation site for p38 MAPK at threonine (Thr)180 in the large subunit of caspase-2 that is conserved among species (Fig. 3a). Caspase-2 was shown to be phosphorylated in Huh7 cells using phos-tag gels and an antibody specific for phospho-Thr180 (Fig. 3b). Stimulation with NGF or pro-NGF increased p-caspase-2 level within hours in these cells (Fig. 3c, d), and reduced using the p38 MAPK inhibitor, SB203580 (Fig. 3e, f). To validate the role of Thr180 in caspase-2, we mutated this site using site-directed mutagenesis, either to alanine (T180A) or to glutamic acid (T180E). Expression of the T180A mutant in Huh7 cells reduced the pro-NGF-mediated increase in LDLR gene expression, while the mutant T180E exhibited no effect (Fig. 3g). In contrast, the mutant T180E slightly elevated LDLR expression in untreated cells (*p* < 0.05) that is in line with it being a functional mimic of Thr180 phosphorylation. As shown by immunoblots, the expression of the mutant T180A reduced the amount of cleaved SREBP2 and caspase-3 induced by pro-NGF in these cells (Fig. 3h). It corroborates previous findings demonstrating that p38 MAPK inhibitors can block the increase in caspase-3 and SREBP2 activation as well as LDLR expression induced by pro-NGF^18^. To assess the association of caspase-3 with caspase-2 we used different caspase-2 mutants in Flag co-immunoprecipitation experiments. Caspase-3 strongly interacted with the Thr180A caspase-2 mutant, indicating that a reduced association of the two is likely mediated by phosphorylation at Thr180 (Fig. 3i). Furthermore, S157A caspase-2 mutant did not bind to caspase-3, nor did the caspase-2 mutant lacking the aminoterminal *Caspase activation and recruitment domain* (CARD) (Fig. 3i). These data show that the CARD domain in caspase-2 is essential for caspase-3 interaction, whereas the Thr180A and S157A caspase-2 mutant constructs behaved differently in binding of caspase-3. Caspase-2 and its phosphorylation at Thr180 is a key event in the regulation of SREBP2 cleavage induced by p75NTRs in Huh7 cells, adding to the complex regulation of SREBPs and its associated genes in hepatocyte cells.

### LDLR is downregulated and Mylip/Idol are upregulated in p75NTR KO mice

**Fig. 4:**
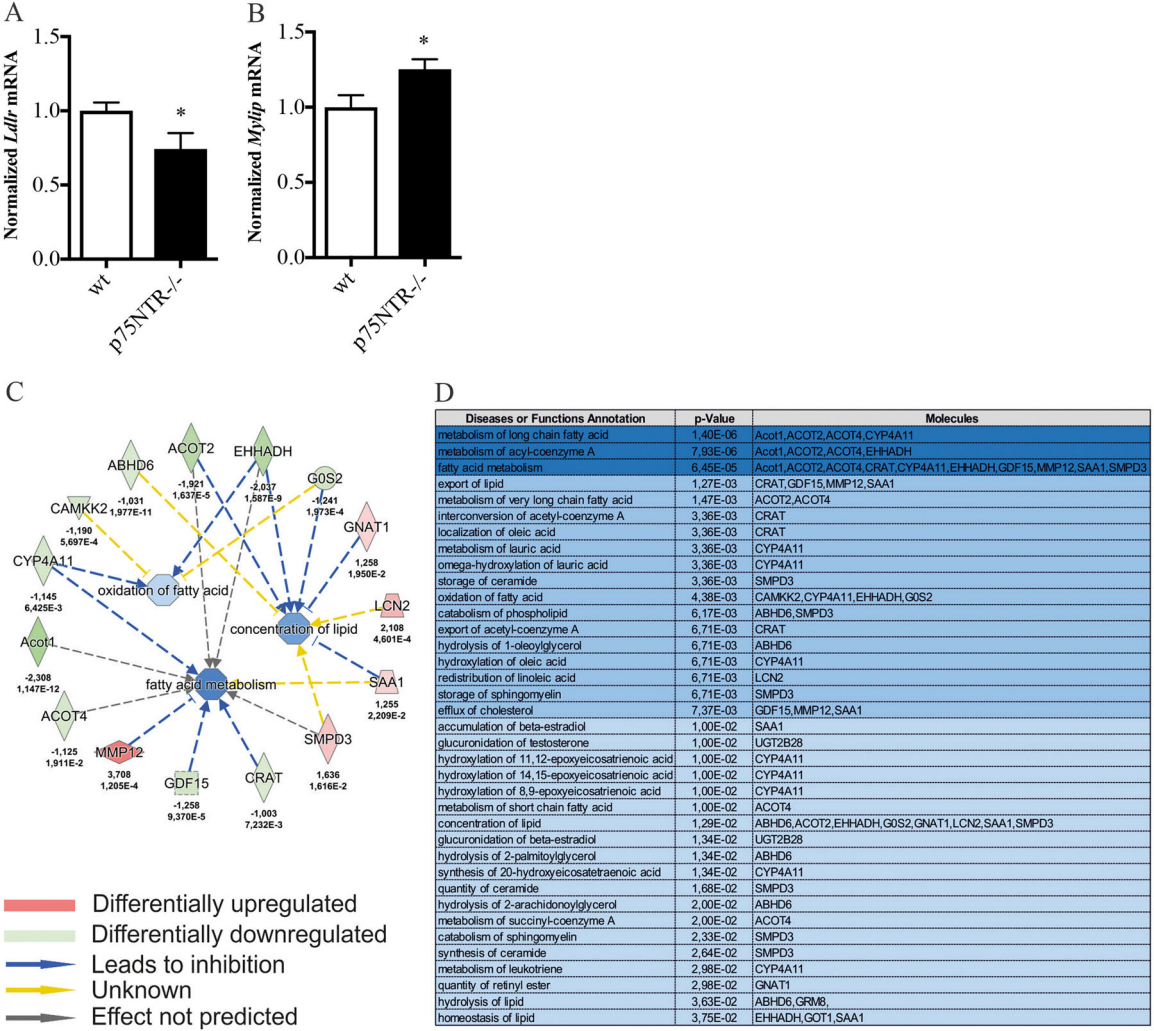
Changes in gene expression in p75NTR gene deleted mouse liver. Gene expression in control and p75NTR knock-out mouse liver was analyzed by qPCR and by using RNA sequencing as described in Materials and methods. **a**, **b** qPCR was done on RNA from wild type (wt) and p75NTR gene-deleted (*−/−*) mouse liver. Results are normalized to *Gapdh* and primers used are shown in Table 2. LDLR expression was reduced in p75NTR-deficient livers compared with controls, while the expression of Mylip/Idol targeting LDLR was increased. Values are mean ± SD, *n* = 3. *p < 0.05 for KO vs. wt liver. **c** RNA-seq followed by Ingenuity Pathway Analyses revealed a network of genes involved in lipid metabolism that are altered in p75NTR-deficient livers compared with controls. Results are expressed as log2 fold changes in expression. The degree of change is indicated with the corresponding adjusted *p* values. The downregulated genes are shown in green and upregulated ones in red. The arrows indicate the directionality of changes in relation to lipid metabolism. The genes shown are: *Acot 1,2,4*, acyl-CoA transferase 1, 2 and 4; *Abhd6*, abhydrolase domain containing 6; *Camkk2*, calcium/calmodulin dependent protein kinase kinase 2; *Cyp4a11*, cytochrome P450 family 4 subfamily A member 11; *Ehhad*, enoyl-CoA hydratase and 3-Hydroxyacyl CoA dehydrogenase; *Crat*, carnitine *O*-acetyltransferase; *Gdf15*, growth differentiation factor 15; *Gnat1*, G protein subunit alpha transducin 1; *Gos2*, G0/G1 switch regulatory protein 2; *Lcn2*, lipocalin 2; *Mmp12*, matrix metallopeptidase-12; *Saa1*, serum amyloid A1; *Smpd3*, sphingomyelin phosphodiesterase 3. **d** Functional annotation of differentially expressed genes between control and p75NTR-deficient mouse liver. An arbitrary cut-off value of 1.5-fold change was used to categorize the genes into functional groups with the adjusted *p* values given in the middle column. Molecules affected are shown in the right column, and their known functions or perturbed pathways are depicted in the left column

We then investigated whether p75NTR plays a role in SREBP and lipogenic gene expression in vivo. To accomplish this, we compared liver tissue from KO and control mice using qPCR. There were no significant changes in the amount of SREBP2 cleaved in the p75NTR KO mice compared with controls (not shown). In contrast, the expression of LDLRs in the livers of p75NTR KO mice (Fig. 4a) was lower along with an increase in the E3 ubiquitin ligase Mylip/Idol for LDLRs^20,21^ that targets LDLR for degradation (Fig. 4b). The reduced levels of LDLRs in p75NTR KO mice may reflect both a reduction in gene expression and an enhanced degradation of LDLRs secondary to Mylip/Idol.

### Gene profiling shows distinct changes in p75NTR-deficient mouse liver

To reveal the pattern of liver genes that are altered in p75NTR gene-deleted mice we employed RNA-seq. Differentially expressed genes (DEG) were further annotated and analyzed using bioinformatic tools. Figure 4c shows a number of DEG in p75NTR KO livers, with the degree of relative change and *p*-adjusted values indicated. The DEG encode proteins linked to the regulation of lipid metabolism, including oxidation and metabolism of fatty acids, and the concentration and synthesis of lipids (Fig. 4c). The expression of lipid-associated genes was generally downregulated in p75NTR-deficient livers as compared with controls as shown in Fig. 4c, d. In addition, we observed increases in genes encoding enzymes involved in biosynthesis of specific classes of lipids, such as that for steroids Hydroxy-delta-5 steroid dehydrogenase (*HSD3B1*), and that for ceramide Serine palmitoyl transferase (*SPTSSB)* (not listed in Fig. 4). In contrast, other genes, such as those encoding Matrix metallopeptidase-12 (*MMP12*) and Serum amyloid-A1 (*SAA1*), were increased in the p75NTR KO mice compared with controls (Fig. 4c).

### Lipid-associated genes are altered in human hepatocyte cells lacking p75NTR

**Fig. 5:**
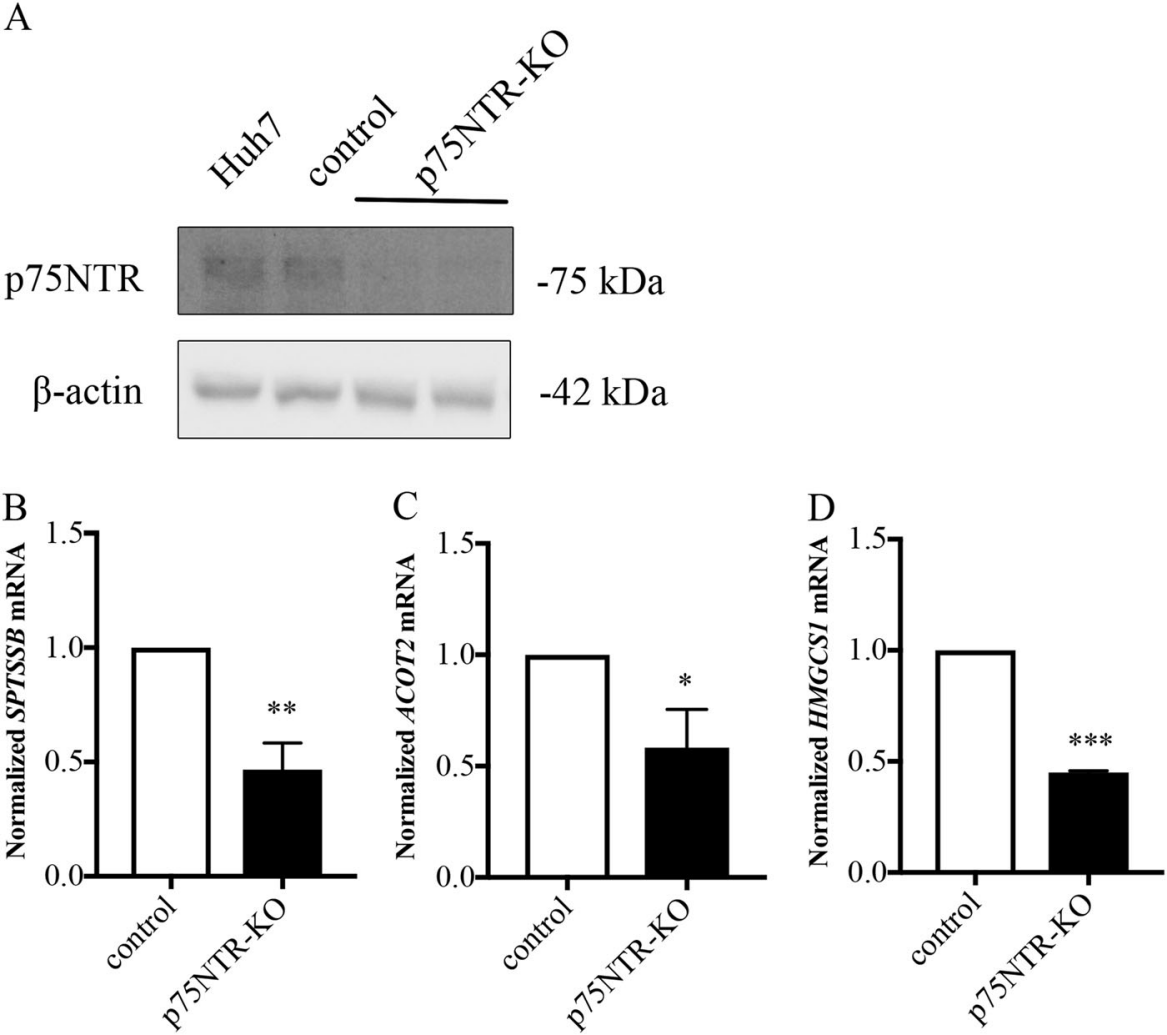
Downregulation of p75NTR by CRISPR/Cas9 in Huh7 cells. The p75NTR gene was inactivated in Huh7 cells by using the CRISPR/Cas9 method as described in Materials and methods. **a** Immunoblot demonstrates a lack of expression of p75NTR in these cells. Genes involved in regulation of lipid metabolism were analyzed using qPCR (**b**–**d**). Primers are shown in Table 3. The gene expression was normalized to *GAPDH* and controls set to 1. Values are mean ± SD, *n* = 3. **p < 0.001 or **p < 0.01 for KO cells vs. controls. **b** Serine palmitoyl transferase (*SPTSSB*). **c** Acyl CoA transferase 2 (*ACOT2*). **d** 3-hydroxy-3-methylglutaryl coenzyme A synthase (*HMGCS1*)

To demonstrate whether the identified genes are similarly affected in human cells, we downregulated p75NTR in human Huh7 hepatocyte cells using the CRISPR/Cas9 method (Fig. 5a). qPCR showed that genes for *SPTSSB* and *ACOT*, linked to level of free fatty acid and acyl-Coenzyme A, were downregulated in their expression in p75NTR-deficient Huh7 cells as compared with controls (Fig. 5b, c), along with 3-hydroxy-3-methylglutaryl coenzyme A synthase (*HMGCS1*) that was twofold downregulated in these cells (Fig. 5d). This suggests that p75NTR signaling most likely influences the mevalonate pathway in cholesterol biosynthesis in these cells.

**Fig. 6:**
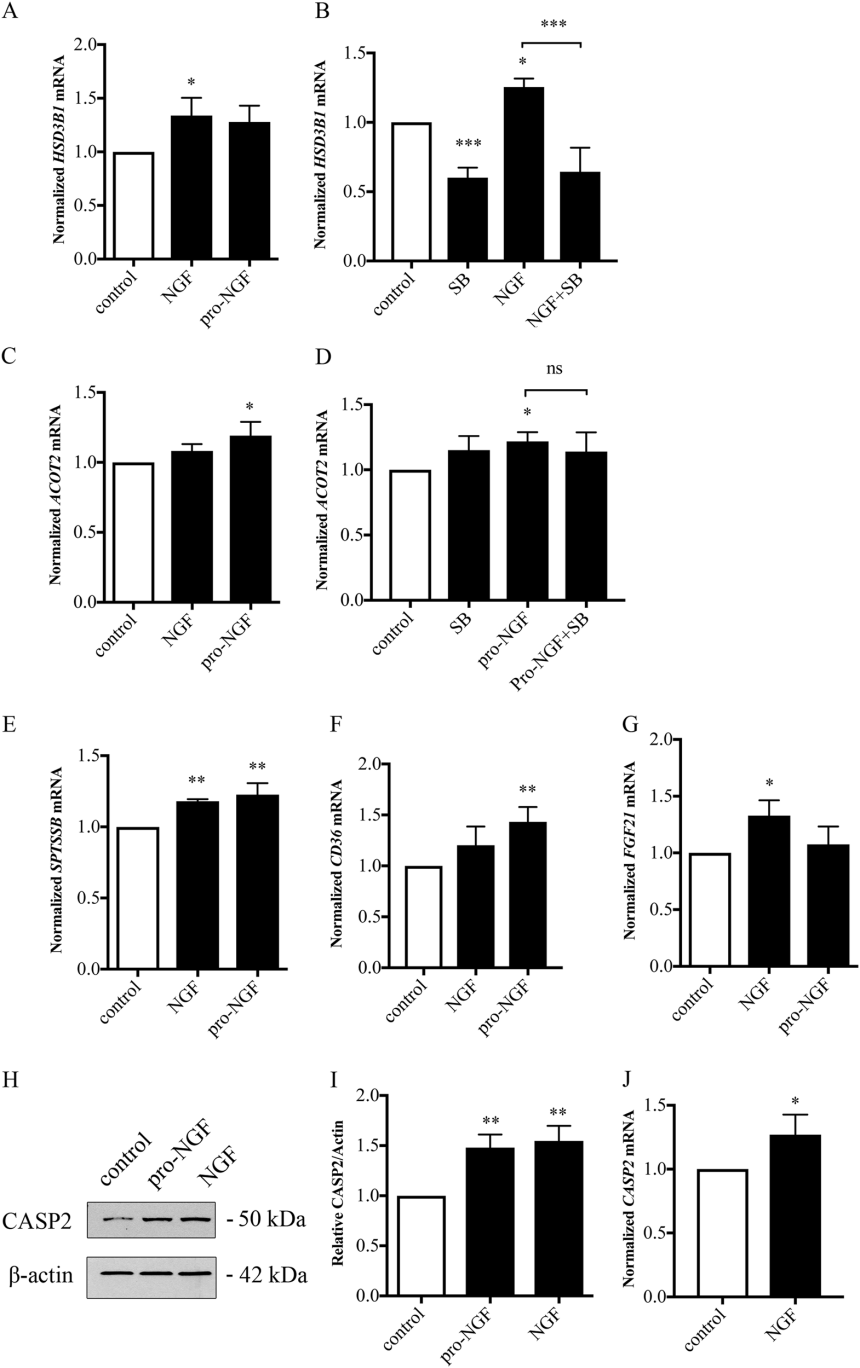
Effect of p75NTR stimulation on lipid-associated genes in Huh7 cells. Huh7 cells were stimulated with 100 ng/ml NGF or 20 ng/ml pro-NGF for 48 h, and the expression of lipid-associated genes was analyzed by qPCR. Primers are shown in Table 2. Results were normalized to *GAPDH* and controls set to 1. Values are mean ± SD, *n* = 3-4. ***p < 0.001, **p < 0.01, or *p < 0.05 for treated cells vs. corresponding controls. **a** Hydroxy-delta-5 steroid dehydrogenase (*HSD3B1*) and **b** in the presence of 1 μM inhibitor, SB203580 (SB). **c** Acyl CoA transferase 2 (*ACOT2*) and **d** in the presence of 1 μM SB. **e** Serine palmitoyl transferase (*SPTSSB*). **f** Cluster of differentiation (*CD36*). **g** Fibroblast growth factor-21 (*FGF21*). **h**–**j** Immunoblots (**h**) with quantification (**i**) showed an increase in caspase-2 (CASP2) expression level by NGF and pro-NGF. **j** qPCR analysis for pro-NGF demonstrated an increase in caspase-2 expression. Values are mean ± SD, *n* = 3. **p < 0.01 or *p < 0.05 for treated cells vs. controls

To corroborate these findings, we stimulated Huh7 cells with NGF or pro-NGF based on the assumption that the downregulated genes in the p75NTR-deficient cells would be upregulated by the neurotrophins. The expression of *HSD3B1* and *SPTSSB* was increased in Huh7 cells by NGF (Fig. 6a, e), whereas that of *ACOT2* was elevated largely by pro-NGF (Fig. 6c). The expression of the cluster of differentiation 36 (*CD36*) involved in fatty acid uptake and metabolism was also increased by pro-NGF (Fig. 6f). Likewise, the expression of the endocrine growth factor, *FGF21* influencing lipid metabolism was increased by NGF in Huh7 cells (Fig. 6g). Moreover, the expression of caspase-2 involved in p75NTR signaling was elevated by the neurotrophins in the Huh7 cells both at the RNA and protein levels (Fig. 6h–j). In contrast, other genes such as those for *ACC1, SCD1*, and *FASN* showed no statistically significant changes in NGF or pro-NGF-treated Huh7 cells (not shown). To assess whether the neurotrophin-mediated increases in lipidogenic genes involved p38 MAPK signaling, we employed the inhibitor, SB203580 (SB), which blocked the increase in *HSD3B1* brought about by NGF (Fig. 6b). In contrast, SB slightly increased the basal expression of *ACOT2* in Huh7 cells and did not further affect the pro-NGF mediated increase of this gene (Fig. 6d). Taken together, these results show that modulation of p75NTR signaling can influence a network of lipid-associated genes in Huh7 cells in a complex fashion with involvement of p38 MAPK and possibly also other signaling mediators requiring further studies.

### Serum lipid levels in p75NTR gene-deleted mice

To investigate whether the changes observed in lipogenic genes in the p75NTR KO mice may affect circulating lipids, we measured serum cholesterol and triglycerides levels. In the homozygous p75NTR-deficient mice the serum levels of cholesterol and triglyceride were significantly reduced compared with controls. However, in the heterozygous p75NTR mice, there was no clear reduction in serum cholesterol showing that the complete absence of this receptor is required for the effects to be discernible (Table 1).

**Table 1:** Serum levels of cholesterol and triglycerides in wild type and p75NTR-deficient mice

Serum	Wildtype (wt), *n* = 7	Heterozygous, *n* = 6	Homozygous (KO), *n* = 11	Difference KO vs. wt, *p*-value
Cholesterol (mmol/l)	3.77 ± 0.84	3.50 ± 0.53	2.91 ± 0.48	*p* = 0.026
Triglyceride (mmol/l)	1.82 ± 0.44	1.51 ± 0.44,50	1.30 ± 0.36	*p* = 0.044

Serum lipid levels were determined as described in Materials and methods. Statistical analysis was done using one-way ANOVA and Bonferroni post hoc test, and using the SPSS program. *P* values <0.05 were considered statistically significant

## Discussion

The pathogenesis of fatty liver is a complex process affected by several factors, such as dietary lipids and sugars, various hormones and cytokines, tissue inflammation, oxidative and ER stress^2–5^, as well as genetic factors^22^. In this work, we provide evidence that the neurotrophins, NGF and its pro-form, pro-NGF are increased in fatty liver that also express the common neurotrophin receptor, p75NTR. In humans, an increase in blood levels of NGF is associated with obesity and the metabolic syndrome in women, but the mechanisms remain unclear^12^. We demonstrate here that deletion of p75NTR influences a network of liver genes involved in lipid synthesis and metabolism. These findings add to the known functions of neurotrophins in influencing the neuronal development, survival, and cell differentiation of the nervous system^23,24^. Moreover, we show here that caspases, particularly caspase-2 plays are important for p75NTR signaling, and in the regulation of SREBP2 cleavage leading to a significant increase in expression of lipid genes.

### NGF and p75NTRs in liver diseases and inflammation

NGF is a classical neurotrophic factor that interacts with the high-affinity tyrosine kinase receptor, TrkA expressed by nerve cells but that can also bind to p75NTR. In contrast, pro-NGF binds preferentially to p75NTR, thereby activating signal pathways partly different from those of TrkA^9,10^. We observed that liver cells express mainly the p75NTR that is in accordance with previous studies showing expression of p75NTRs in various peripheral organs, including muscle and adipocyte tissue^6,25,26^.

The presence of NGF in serum from patients with inflammatory diseases was previously determined by a sensitive enzyme immunoassay^27,28^. In the liver, NGF is localized to hepatocytes and Kupffer cells (liver macrophages). In line with this, NGF is synthesized by macrophages after nerve injury^29^. In control liver, the levels of NGF levels are low but are increased in hepatocarcinoma^30^, signifying its role in tumor cell survival or proliferation. NGF is also upregulated in cholestatic livers and protects hepatocytes against oxidative stress^13^. NGF and pro-NGF levels were increased in fatty livers of leptin-deficient *ob/ob* mice^8^, and as shown here also in *db/db* mice that lack the leptin receptor (Fig. 1). In addition, p75NTR was expressed in the fatty liver, suggesting a role of p75NTR signaling in lipid metabolism. We have recently reported that stimulation of p75NTRs by NGF influenced SREBP, LDRL levels, and lipoprotein uptake in Huh7 cells^8^.

### Regulation of SREBP pathway by caspases

Cellular caspases play an important role in regulation of SREBP2 and LDLR expression downstream of p75NTR signaling. Particularly, p38 MAPK was activated by NGF/pro-NGF leading to an increased phosphorylation of caspase-2 (Fig. 2). A change in the phosphorylation of caspase-2 likely affects its binding of other molecules, such as caspase-3. Along with this, we previously reported that the caspase-2/caspase-3 interaction was reduced upon activation of p38 MAPK by p75NTR^8^. In SREBP2, there is a caspase-3 cleavage site at amino acid 468 in the cytosolic part of SREBP2 with all domains required for its activity as a transcription factor. NGF can increase LDLR expression in Huh7 cells that depend on SREBP2 and functional SRE-binding elements in the LDLR promoter^8^. Using the compound, PF429242 (ref. ^18^), we obtained evidence that the caspase-3 induced SREBP cleavage is distinct from the S1P/S2P pathway activated by the low cell cholesterol level. However, it is likely that the p75NTR-mediated pathway for SREBP regulation may functionally interact with the classical one, particularly under cell stress conditions and after lipid accumulation such as in fatty liver. Regarding the cellular site of SREBP2 activation, it has been shown that a fraction of caspase-2 in the cells is located adjacent to the Golgi compartment^8,31^. This finding suggests that the caspase-3-induced cleavage of SREBP2 may preferentially occur at this intracellular site following phosphorylation of caspase-2 by p38 MAPK.

### Caspase-2 in lipid metabolism

Caspase-2 is an upstream caspase, which can be activated following cell stress and by some other conditions^32–34^. Caspase-2 has several functions in the cell and among others can influence tumor cells^33,35^, and metabolic pathways including those linked to lipid metabolism^2,4,36^. The promoter region of mouse *caspase-2* gene contains binding sites for SREBP2, and the enzyme can modulate the effects of SREBP2 on lipogenic gene expression^37^. We observed that human caspase-2 expression was increased by NGF/pro-NGF in Huh7 cells, implying a feed-forward loop involving caspase-2 in modulation of SREBP2 and lipid gene expression. Caspase-2 can also directly cleave the enzyme S1P leading to an activation of SREBP1/2 in the ER membrane under conditions of fatty liver^38^. We show here that the pro-NGF-mediated cleavage of SREBP2 occurred also in the presence of PF-429242 inhibiting the S1P enzyme (Fig. 2). Together, these emphasize the complex mode of SREBP regulation under normal and diseases conditions.

We show here that caspase-2 is phosphorylated in Huh7 cells at a novel Thr180 site, in the large subunit contributing to the regulation of SREBP2. Previous studies performed in oocytes showed that caspase-2 can be phosphorylated at serine residue 340 in the linker region, resulting in attenuation of caspase-2 activity in cell death^32^. Likewise, phosphorylation of serine 157 in caspase-2 influenced its function in caspase-8-mediated apoptosis^39^. We observed that the S157A mutant did not bind to caspase-3, in contrast to the Thr180A mutant or to wild-type caspase-2. It remains to be shown whether there is an interplay between phosphorylation of different residues in caspase-2 and whether that of Thr180 may affect other activities ascribed to caspase-2 (refs. ^33,34^).

### RNA-seq analyses of genes altered in p75NTR-deficient livers

In addition to SREBP2 (ref. ^8^), we observed that SREBP1 was cleaved by pro-NGF. This suggests that several genes influenced by the SREBP transcription factors can likely be targets for the action of p75NTR signaling in liver. In line with this, gene profiling using RNA-seq demonstrated that many lipid-associated genes were differentially expressed in control and p75NTR-deficient mouse livers, and several have been linked to the SREBP pathway^15^. Bioinformatic analyses revealed that DEG encode proteins forming functional networks involved in the uptake (LDLR), storage, and oxidation (Acot1, Acot-2, Acot-4, Ehhadh) of various lipids in the cell. Data obtained confirmed that these genes were also downregulated in p75NTR-deficient Huh7 cells pointing to a similar gene regulation in mouse and human cells by p75NTR signaling. The downregulated genes in Huh7 p75-deficient cells included *SPTSSB, ACOT2*, and *HMGCS1* as shown by qPCR (Fig. 5). SPTSSB is a key enzyme in ceramide biosynthesis, while HMGCS1 in the mevalonate pathway for cholesterol biosynthesis. To study whether the lipidogenic genes are directly modulated by the neurotrophins, the Huh7 cells were stimulated with NGF or pro-NGF followed by qPCR analyses. Several genes were upregulated by the neurotrophins including *ACOT2, CD36, HSD3B1*, and *SPTSSB* (Fig. 6). To investigate whether p38 MAPK is involved in their regulation we employed the inhibitor SB203580, showing a differential response with some genes downregulated (*HSD3B1*) while others not being affected. This shows that in addition to the p38 MAPK/SREBP2 pathways other factors are also involved in their gene regulation.

Among the gene analyzed, we were particularly interested in *LDLR* that was reduced in p75NTR KO mice as compared with controls. This finding confirms that p75NTR signaling is able to influence LDLR levels in vivo. Furthermore, the expression of the E3 ubiquitin ligase Mylip/Idol that targets LDLR for ubiquitin-mediated degradation^20,21^ was elevated in the p75NTR KO mice. This suggests that the rate of degradation of LDLRs is increased in the p75NTR KO mice. Our previous studies showed that p75NTRs can increase LDLRs and reduce Mylip/Idol in hepatocyte cells^8^ and in primary neurons^40^. Results further revealed that the expression of *Fgf21* (ref. ^41^) was reduced in p75NTR-deficient mice compared with controls. FGF21 has several important biological functions in the body, influencing glucose metabolism^42^, hepatic metabolism^43^, and uptake of lipoproteins via LDLRs^44^. FGF21 also counteracts fatty liver and enhances insulin sensitivity in obese mice with an increase in energy utilization^42^. The precise role of FGF21 in p75NTR-associated changes in lipid metabolism warrants further studies.

### p75NTR in caspase-2 regulation and metabolism

**Fig. 7:**
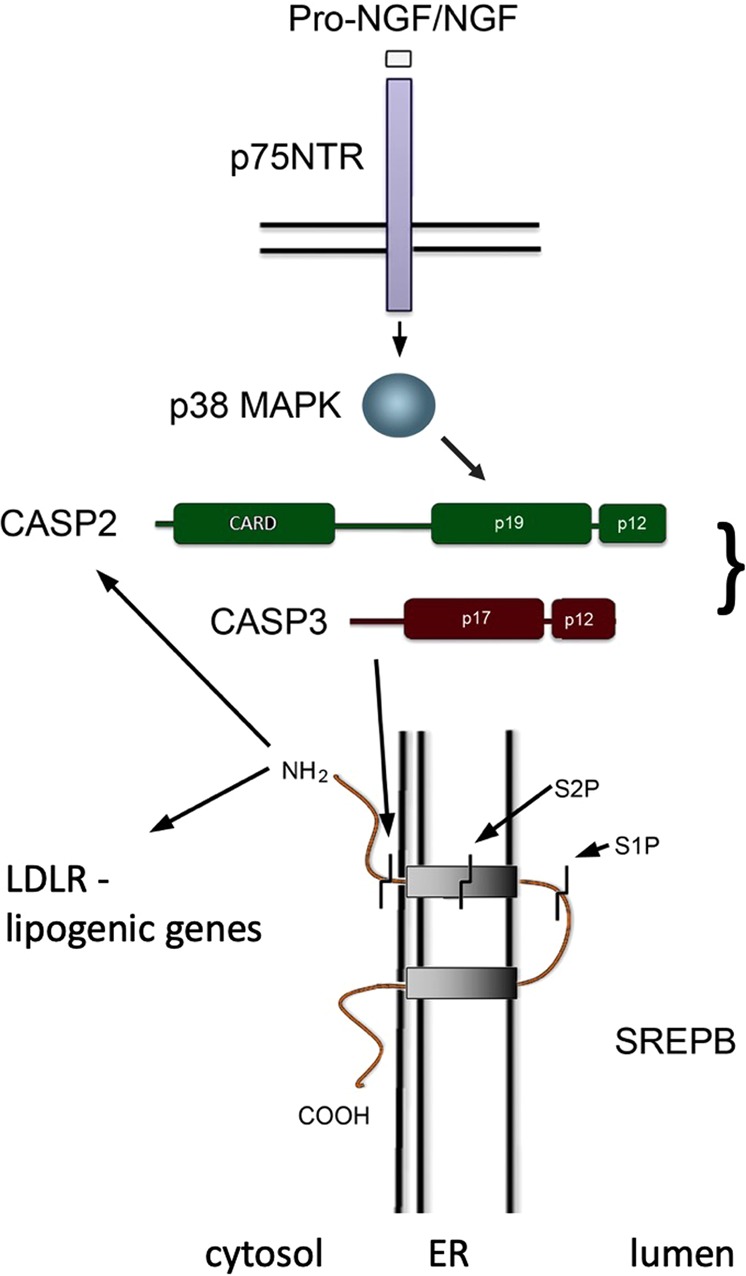
Schematic view of the role of caspase-2 in p75NTR signaling and lipid metabolism. We propose the following scheme for the action of p75NTR and of caspase-2 in SREBP2 and associated gene regulation. Stimulation of p75NTR by NGF or pro-NGF activates the signaling protein p38 MAPK that can phosphorylate caspase-2 (CASP2) leading to the release of caspase-3 (CASP3) from its complex. CASP3, in turn, can cleave SREBP molecules to yield an active transcription factor. SREBP responsive genes include LDLR and other lipogenic genes but also CASP2 itself. Normally SREBP is processed by the enzymes S1P and S2P via a feedback inhibition determined by the cell cholesterol level. We hypothesize that the steroid-mediated and neurotrophin/p75NTR-induced SREBP pathways are distinct but functionally linked

Recent studies have revealed an important role of caspase-2 in models of fatty liver disease and in lipoapoptosis^2,4,19,38^. Our results (Fig. 7) show that p75NTR signaling plays an essential role in regulation of caspase-2 and lipid metabolism. These data support recent results indicating that the p75NTR-deficient mice are protected against diet-induced fatty liver disease with an improved insulin sensitivity and energy balance^6,7^. These effects were ascribed to alterations in adipocyte cells and in energy expenditure evoked by the lack of p75NTRs. We show here that several genes involved in lipid pathways are altered in livers of p75NTR-deficient mice, which probably contributes to general metabolism and improved metabolic status of the mice. Levels of major serum lipid classes, cholesterol and triglycerides, were significantly reduced in homozygous p75NTR-deficient mice compared with controls. In heterozygous p75NTR mice, however, there was no reduction in serum lipids (Table 1) demonstrating that the complete lack of p75NTRs is necessary to evoke these effects.

Increased serum cholesterol and triglyceride levels are risk factors for metabolic and cardiovascular diseases in man and can be treated with drugs like statins, which also have side effects^45–47^. Monoclonal antibodies against PCSK9 have shown potential in clinical trials for familiar hypercholesterolemia and cardiovascular diseases^48^. Recent studies targeting caspase-2 by either gene ablation or using pharmacological inhibition demonstrated beneficial effects against diet-induced fatty liver in mice^38^. Our results suggest that modulation of p75NTR signaling^49^ could target lipid and metabolic disorders. It will be important to study changes in gene expression in other tissues such as brain in the p75NTR KO mice and whether these can contribute to or influence lipid-associated neurological disorders.

## Materials and methods

### Vector constructs and materials

Wild type and mutant LDLR promoter firefly luciferase reporter plasmids were from Addgene (numbers 14940 and 14945) as well as Flag-tagged Caspase-2 plasmid (number 11811), C303A mutant caspase-2 (number 10812), and CARD domain mutant caspase-2 (number 10810) were from Addgene. Threonine180 mutant caspase-2 (Thr180A) and (Thr180E) constructs as well as serine139 mutant (S139A) were generated using the Quickchange Lightning site-directed mutagenesis kit (Agilent) and was confirmed by sequencing. A phospho-specific antibody against threonine residue 180 (Thr180) in caspase-2 was commercially made (Eurogentec, Liege, Belgium). This antibody detected a single band in Huh7 cells stimulated with NGF and pro-NGF. Specificity of the antibody towards phospho-Thr180 caspase-2 was further tested using indirect ELISA against native and modified peptide (Eurogentec). Sources of other antibodies and chemicals are specified below under the appropriate headings.

### Animal experiments

All animal procedures were approved by the ethics committee and carried out in accordance with the European Communities Council Directive (86/609/EEC). The mice were maintained in a temperature- and light-controlled environment and received a standard diet. p75 neurotrophin receptor gene deleted (p75NTR KO) mice^50^ were obtained from The Jackson Laboratory (Bar Harbor, ME USA) and bred in the laboratory to produce wild type, heterozygous, and homozygous KO mice. Genetically obese leptin-deficient *ob/ob* mice (The Jackson Laboratory) and leptin-receptor-deficient *db/db* mice were used as a model for fatty liver. Liver tissue was homogenized, and an equal amount of protein was subjected to immunoblotting using anti-NGF (1:1000; Alamone Labs, Jerusalem, Israel), anti-pro-NGF antibodies (1:300; Alamone Labs), and other antibodies as described below. For immunostaining paraffin sections were made from control (heterozygous) and homozygous (*db/db*) mice and processed for immunostaining essentially as described^51^. In short, sections were dewaxed using xylene and rehydrated in decreasing concentrations of ethanol, and antigen retrieval was done by boiling in 10 mM citrate buffer, pH 6.0 followed by washing with phosphate-buffered saline (PBS), inactivation of endogenous peroxidase for 15 min, and blocking in 5% normal horse serum in PBS-Tween 20 (0.5%) buffer for 1 h at room temperature (RT). Anti-NGF antibody (diluted at 1:50) was added overnight at +4 °C, sections washed, and biotinylated horse ant-rabbit secondary antibody (1:700) was added for 2 h at RT. Sections were again washed and the immunostaining visualized using the Vectastain ABC kit PK-6100 Elite (Vector Laboratories, Burlingame, CA, USA) following the instructions by the vendor, mounted, and representative images were taken using the Zeiss fluorescent microscope.

### Cell culture and transfections

Human Huh7 hepatocyte cells were cultured in minimum essential medium Eagle (MEM, Sigma) supplemented with 10% fetal calf serum (Gibco/Life Technologies, Inc., Paisley, UK) at 37 °C in 5% CO_2_. Cells were transfected with 2–4 μg of expression plasmids using Transfectin or FUGENE reagents (Bio-Rad, Espoo, Finland) for 24 h or 48 h (ref. ^8^). Cells were stimulated with different concentrations of NGF or pro-NGF (cleavage-resistant, mutant protein) (Alamone Labs) for various periods of times. In some experiments, 0.5 μM PF429241 (PF, Sigma) was added to inhibit the site-1 protease (S1P) to distinguish between S1P and caspase-3 in the regulation of SREBP.

### Establishment of p75NTR gene-deficient cells using CRISPR/Cas9

Gene editing of p75NTR using the Clustered regularly interspaced short palindromic repeats (CRISPR) Cas9 endonuclease (CRISPR-Cas9) system was performed as described^52^. Guiding RNAs (gRNAs) to target p75NTR were designed using the CRISPR design tool (http://crispr.mit.edu) and are listed in Table 2. They were further cloned into the pSpCas9(BB)-2A-GFP vector (Addgene, px458, number 48138) using FastDigest BpiI (Thermo Scientific) and T4 DNA ligase (New England Biolabs). Huh7 cells were cultured in 24-well plates and transfected with 500 ng DNA and using polyethylenimine (PEI) reagent (Polysciences, Warrington, USA) in a ratio of 1 to 3. Cas9-control cells were transfected with plasmid without the specific gRNAs. One day after transfection, cells were sorted using FACSAriaII (BD Biosciences) at the Biomedicum Flow Cytometry Core Facility (Helsinki, Finland). Single-cell clones were grown in 96-well plates to generate stable cell lines that were grown further. p75NTR levels were analyzed using immunoblotting.

**Table 2:** Guide RNA and guide RNA primer sequences

gRNA	Strand	Sequence
guide p75NTR	+	AAAGCCTGCAACCTGGGCGA
gRNA primers		
guide p75NTR_F		5′-caccgAAAGCCTGCAACCTGGGCGA-3′
guide p75NTR_R		5′-aaacTCGCCCAGGTTGCAGGCTTTc-3′

### Immunoblotting

Tissue and cells were lysed in a buffer containing 150 mm NaCl, 1 mM EDTA, and 50 mM Tris-HCl, pH 7.4, supplemented with 1% Nonidet P-40, 0.25% sodium deoxycholate, 1% SDS, and protease inhibitors^51,53^. Protein concentrations were determined using the BCA protein assay (Thermo Scientific), and an equal amount of protein per sample was subjected to SDS-PAGE, and blotted onto nitrocellulose filters (Amersham Biosciences, Helsinki, Finland). The filters were incubated for 1 h in 50 mM Tris-HCl, pH 7.5, 150 mM NaCl, supplemented with 0.1% Tween 20, and 5% skimmed milk or 5% BSA at room temperature and then overnight at 4 °C with primary antibodies as follows: anti-NGF (1:1000; Alomone Labs, AN-240), anti-Pro-NGF (1:500; Alomone labs, ANT-005), anti-p75NTR intracellular region (1:5000; Millipore, 07-476), anti-p75NTR (1:1000; Cell Signaling, 8238T), anti-Caspase-2 (1:1000; Enzo Life Sciences, 11B4), anti-phospho-Caspase2 (1:200; Eurogentec), anti-Caspase 3 (1:1000; Cell Signaling, 9665), anti-cleaved-Caspase 3 (1:1000; Cell Signaling, 9664), anti-SREBP2 Carboxyl terminal region (1:500; BD Biosciences, 557037), anti-SREBP1 (1:1000; Novus Biologicals, NB600-582), anti-Flag (1:2000; Sigma, F1804), and anti-β-actin (1:10,000; Sigma). After washing, the filter was incubated with horseradish peroxidase-conjugated secondary antibodies (1:2500; Jackson ImmunoResearch, Espoo, Finland), followed by detection using enhanced chemiluminescence (Thermo Scientific, Finland). Quantification was performed using ImageJ.

### Immunoprecipitation

Immunoprecipitation was done using cell lysates from Flag-tagged wild-type and mutant caspase-2-expressing Huh7 cells. In brief, RIPA lysis buffer containing 150 mM NaCl, 1% Triton X-100, 0.5% sodium deoxycholate, 50 mM Tris-HCl, and 0.1% SDS, pH 8.0, supplemented with protease inhibitor mixture (Roche Applied Science) was added, and preclearing was done followed by an overnight incubation with anti-Flag antibodies (Sigma). Protein G-agarose (Roche Applied Science, Germany) was added and incubated in a rotor at 4 °C for 2 h, and the agarose beads were collected by centrifugation, washed three times, and boiled in loading buffer, and the immunoprecipitates were analyzed by immunoblotting using anti-caspase-3 and anti-Flag antibodies.

### Phos-tag gel analysis

Huh7 cells were lysed in RIPA buffer containing protease inhibitors and phosphatase inhibitors cocktail (Roche). Samples were subjected to 8% SDS-PAGE gels or in-gel combination of 100 μM Phos-Tag^TM^ (Wako Chemicals) with 400 μM MnCl_2_ (ref. ^54^). After running, the gel was equilibrated for 10 min in transfer buffer supplemented with 5 mM EDTA and transferred to nitrocellulose filters as described above.

### Gene promoter assay

Cells were transfected for 24 h with the plasmids encoding wild type or mutant (lacking the SREBP2-responsive element) LDLR promoters upstream of firefly luciferase. As a control for transfection efficiency, we used the Renilla luciferase pRL-TK vector (Promega). Cells were stimulated with 50 ng/ml NGF, 5 ng/ml pro-NGF, or 1 μM simvastatin for 24 h followed by measurement of firefly and Renilla luciferase activities using a luminometer (Promega, Biofellows, Helsinki, Finland) as described^8,44^. Results are shown as fold increase in firefly luciferase activity normalized to that of Renilla luciferase.

### RNA isolation and quantitative PCR

RNA was extracted from control and NGF-treated Huh7 cells using the RNeasy mini kit (Qiagen) followed by cDNA synthesis using SuperScript VILO cDNA synthesis kit (Invitrogen). LightCycler 480 SYBR Green I MASTER (Roche Applied Science) real-time quantitative PCR assays were performed using LightCycler 480 (Roche Applied Science) with a 96-well block as described^44,55^. Initial incubation was at 95 °C for 15 min, followed by 40 cycles at 95 °C for 15 s, 60 °C or 63 °C for 20 s and 72 °C for 10 s. Each sample was done in triplicates and the experiments were repeated three times and normalized to glyceraldehyde-3-phosphate dehydrogenase (*GAPDH)* using the ΔΔCt (threshold cycle) method. The amplified product was checked by melting curve analysis spanning the temperature range from 65 °C to 95 °C with a ramping rate of 0.03 °C/s, and further confirmed by 2% agarose gels electrophoresis. qPCR was performed using the primers shown in Table 3.

**Table 3:** List of primer sequences

Gene	Forward primer 5′–3′	Reverse primer 5′–3′
*HSD3B1*_human	TCTTCGGTGTCACTCACAGAG	GGCACACTAGCTTGGACACA
*SPTSSB*_human	ATTTGAGGCGTGTGAAGGAA	GCACAGCAGCTAATGATTTGG
*FGF21*_human	CCAAGAGGTGGTTTTTCCAG	ACCTGGGGTCCTTTCAATG
*ACOT2*_human	AGTGGAGGTTTCAACACAGGA	GGGCAGAGCTGTCTGCTAAC
*HMGCS1*_human	CATTAGACCGCTGCTATTCTGTC	TTCAGCAACATCCGAGCTAGA
*CASP2*_human	CAAGAAGCTCCGCCTGTC	ATTCAGGAGTGCAAGGCTTC
*CD36*_human	AAGCCAGGTATTGCAGTTCTTT	GCATTTGCTGATGTCTAGCACA
*GAPDH*_human	TTCGTCATGGGTGTGAACCA	CTGTGGTCATGAGTCCTTCCA
*Mylip*_mouse	TGTGGAGCCTCATCTCATCTT	AGGGACTCTTTAATGTGCAAGAA
*Ldlr*_mouse	GCATCAGCTTGGACAAGGTGT	GGGAACAGCCACCATTGTTG
*Gapdh*_mouse	GGGTTCCTATAAATACGGACTGC	CCATTTTGTCTACGGGACGA
*FASN*_human	AAGGACCTGTCTAGGTTTGATGC	TGGCTTCATAGGTGACTTCCA
*ACC1*_human	CTGTTGGCTCAGATACACTC	GCCACAGTGAAATCTCGTT
*SCD1*_human	TCACTTTGATTCCTACCTGCAA	GACGATGAGCTCCTGCTGTTA

### RNA-Seq library preparation, sequencing and data analyses

Total RNA was isolated from livers of control and p75NTR-deficient mice as above and further treated with DNAase (New England Biolabs) to remove DNA contamination. Sample RNA was analyzed for integrity and quality on Agilent Bioanalyzer 2100 (Agilent Technologies Inc., Santa Clara, CA, USA). NEBNext Ultra Directional RNA Library Prep Kit for Illumina was used to generate cDNA libraries for next-generation sequencing following the manufacturer´s recommendation. The cDNA libraries were barcoded enabling sample multiplex for sequencing, and library quality was assessed by a Bioanalyzer (Agilent DNA High Sensitivity chip) and quantity by Qubit (Invitrogen). Up to six RNA libraries from control and p75NTR mice were multiplexed using the Illumina NextSeq Sequencer System (Illumina, San Diego, CA, USA), and sequenced using 75-bp single-end sequencing chemistry. The sequence data were processed for quality data analysis using the FastQ and Trimmomatic tools, and reads aligned to the GENCODE M12 (GRCm38.p5) mouse reference. STAR-aligner was set to gene level output mode to get sorted alignment files in binary mode^56^. To produce the counts for the samples the feature Counts software was used^57^. The counts formed the basis for calculation of differential expression statistics employing the DESeq2 software in R environment^58^. Data on differential gene expression between control and p75NTR deficient samples are shown as log2FoldChange with Wald test *p* values, and using Benjamini–Hochberg adjusted *p* values. The Ingenuity Pathway Analysis tool was used to functionally characterize the DEG^59^. The complete RNA-seq data sets are available from the Gene Expressing Omnibus database (http://www.ncbi.nlm.nih.gov/geo/) under the accession number GSE122424 (release date 01.05.2019).

### Lipid analyses

Mice were fasted for 4 h and blood samples collected for analyses. Serum triglycerides were determined with the enzymatic method (GPO-PAP 1488872 kit, Roche Diagnostics, Mannheim, Germany), and total cholesterol using the enzymatic method (CHOD-PAP 1489232 kit, Roche Diagnostics) as described previously^60^.

### Statistical analyses

Statistics were done using one-way ANOVA followed by a Bonferroni post hoc test for three or more groups. The Student’s *t*-test was used in experiments with two groups with GraphPad Prism version 7.0 (GraphPad Software, La Jolla, CA). Values are expressed as means ± SD, and *p* ≤ 0.05 was considered as significant.
